# Variation and Evolution in the Glutamine-Rich Repeat Region of *Drosophila* Argonaute-2

**DOI:** 10.1534/g3.116.031880

**Published:** 2016-06-16

**Authors:** William H. Palmer, Darren J. Obbard

**Affiliations:** Institute of Evolutionary Biology and Centre for Infection, Evolution and Immunity, University of Edinburgh, EH9 3FL UK

**Keywords:** Argonaute-2, *Drosophila melanogaster*, RNA interference, repetitive elements

## Abstract

RNA interference pathways mediate biological processes through Argonaute-family proteins, which bind small RNAs as guides to silence complementary target nucleic acids . In insects and crustaceans *Argonaute-2* silences viral nucleic acids, and therefore acts as a primary effector of innate antiviral immunity. Although the function of the major *Argonaute-2* domains, which are conserved across most Argonaute-family proteins, are known, many invertebrate *Argonaute-2* homologs contain a glutamine-rich repeat (GRR) region of unknown function at the N-terminus . Here we combine long-read amplicon sequencing of *Drosophila* Genetic Reference Panel (DGRP) lines with publicly available sequence data from many insect species to show that this region evolves extremely rapidly and is hyper-variable within species. We identify distinct GRR haplotype groups in *Drosophila melanogaster*, and suggest that one of these haplotype groups has recently risen to high frequency in a North American population. Finally, we use published data from genome-wide association studies of viral resistance in *D. melanogaster* to test whether GRR haplotypes are associated with survival after virus challenge. We find a marginally significant association with survival after challenge with *Drosophila* C Virus in the DGRP, but we were unable to replicate this finding using lines from the *Drosophila* Synthetic Population Resource panel.

Argonaute proteins are the effectors of eukaryotic RNA interference (RNAi) pathways, using short nucleic acid guide sequences to target complementary sequences for transcriptional or posttranscriptional repression. RNAi-related pathways mediate a diverse range of biological processes, from regulation of developmental genes through miRNAs and endogenous siRNAs, to defense against genomic parasites such as transposable elements via piRNAs (reviewed in Carmell *et al.* 2002; Meister 2013). RNAi is also a key line of antiviral defense in plants (Lindbo *et al.* 1993; Ratcliff *et al.* 1997), fungi (Segers *et al.* 2006), ecdysozoan animals such as arthropods and nematodes (Wilkins *et al.* 2005; Wang *et al.* 2006), and possibly even in some vertebrate tissues (Li *et al.* 2013; Maillard *et al.* 2013). In insects, antiviral RNAi is mediated by an RNA Induced Silencing Complex that contains Agonaute-2 (Ago2). This complex is guided by 21nt siRNAs ‘diced’ from viral replicative intermediates and other dsRNA substrates by Dicer-2 (Okamura *et al.* 2004; Lee *et al.* 2004; Wang *et al.* 2006) and bound to Ago2. Ago2 then uses these siRNAs to target the ’slicing’ of viral single-stranded RNA, rendering the targeted viral genome or transcript nonfunctional.

Despite the diverse biological roles played by Argonaute proteins, their structural organization is generally conserved over deep evolutionary time (Swarts *et al.* 2014). For example, eukaryotic Argonaute proteins have a PIWI domain that binds and/or ‘slices’ target nucleic acids (Song *et al.* 2004; Parker *et al.*, 2004), MID and PAZ domains that bind the 3′ and 5′ ends of the small RNA, respectively (Lingel *et al.* 2003; Ma *et al.* 2004, 2005; Boland *et al.* 2010), and an N-domain which is involved in duplex unwinding (Kwak and Tomari 2012). Nevertheless, in contrast to these highly conserved domains, the N-terminal region of Argonaute proteins tends to be disordered and lack sequence complexity, and is highly variable between species (Hain *et al.* 2010). This variation is particularly striking in the arthropod antiviral gene, Ago2, where the N-terminal region is often composed of numerous glutamine-rich repeat motifs (‘GRR’; Hain *et al.* 2010). For example, even between closely related species such as *Drosophila melanogaster* and *D. simulans*, the N-terminal sequence divergence is extensive. In *D. melanogaster*, Ago2 includes one of the most repetitive amino acid sequences in the genome (Jorda and Kajava 2009), while in *D. simulans* it is markedly different, with only one large duplication of almost the entire N-terminus (Figure 1 and Figure 2).

**Figure 1:**
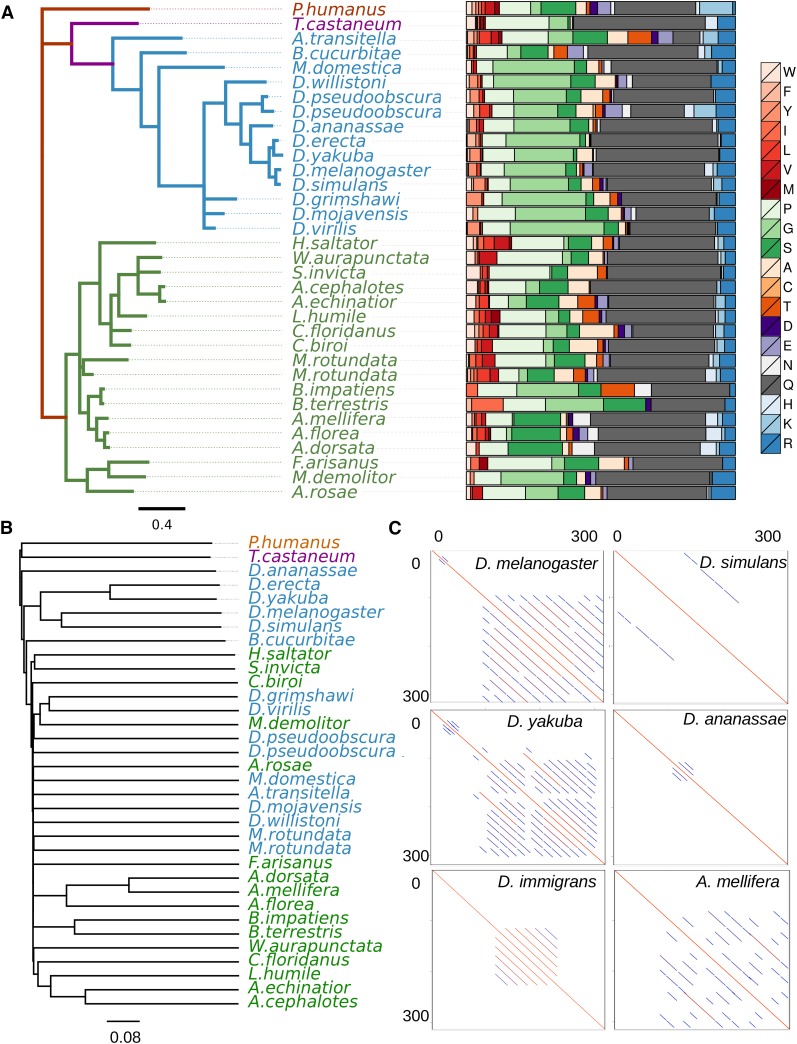
GRR evolves rapidly but maintains similar sequence composition. (A) The gene tree of conserved *Ago2* sequence C-terminal to the GRR, for selected insect species, along with the corresponding amino acid residue composition of the entire GRR for that species. Hymenopteran species are colored green and dipteran species are colored blue. Across the insects analyzed there is conservation of the residues from which the GRR is composed. Amino acid color scheme: WFYILVM (reds, hydrophobic), PGS (greens, small secondary structure breakers), ACT (oranges, small amino acids), DE (purples, larger amino acids), NQ (greys, carboxamide side chains), and HKR (blues, electrically charged side chains). (B) Neighbor joining tree drawn from Frequency feature profile (FFP) clusters derived from the protein sequence of the entire GRR region: the lack of internal resolution reflects the rapid divergence of the GRR among species. (C) The GRR structure can change rapidly among closely related species. Shown are dot-plots for the N-terminal 300 amino acids of Ago2 (plotted against itself) in *D. melanogaster*, *D. simulans*, *D. yakuba*, *D. ananassae*, *D. immigrans*, and *A. mellifera*. In these dot-plots the diagonal line from corner to corner represents the sequence identity to itself, and the successively shorter parallel lines reflect the multiple scales of self-similarity within the sequence (see Figure 2 for alignments).

**Figure 2:**
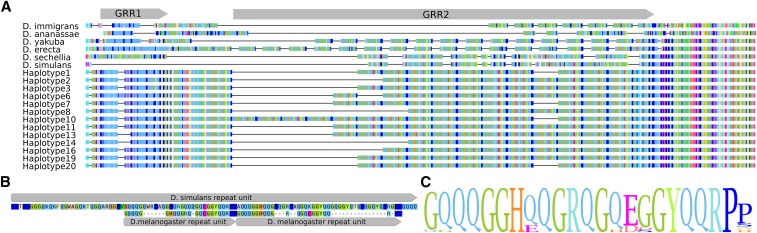
*Drosophila* GRR alignments. (A) Alignments of the GRR among seven Drosophila species for which the complete N-terminal region is available, including a subset of the newly sequenced DGRP haplotypes. Gray arrows above the alignment denote the GRR1 and GRR2 regions that align poorly across species, with the start of the more conserved Ago2 sequence to the right. (B) Alignment between *D. simulans* and *D. melanogaster* GRR repeat units showing how the *D. simulans* repeat motif appears to derive from a pair of neighboring motifs in *D. melanogaster*, and exemplifying the rapid protein evolution of this region. (**C**) A sequence logo built from the alignment of unique repeat units found in *D. melanogaster*. The total height reflects overall diversity, and the height of the letter at each position signifies the frequency of that amino acid across distinct repeat motifs.

In *D. melanogaster*, the GRR region is composed of two distinct repeat regions (GRR1 and GRR2; Hain *et al.* 2010). The most N-terminal, GRR1, is a 6 amino acid imperfect repeat (QQLQQP) present in two to four copies, while GRR2 is a 23 residue imperfect repeat (Figure 2) previously reported to occur between 7 and 11 times in succession in laboratory strains (Hain *et al.* 2010). Although genetic studies have elucidated the function of Ago2 in *D. melanogaster*, the role of the GRR is still unknown. In other proteins, long poly-glutamine-rich regions have been implicated in increased protein adhesion and protein complex formation, and underlie numerous human diseases (reviewed in Fan *et al.* 2014). However these are generally long contiguous tracts of glutamine residues, in contrast to the short complex repeat units observed in the Ago2 GRR. Further, *Ago2* GRR deletions appear to have no effect on RISC assembly in *Drosophila* (Liu *et al.* 2009), suggesting that this domain is not required for binding siRNAs or catalyzing target cleavage.

The absence of known function makes it difficult to predict which evolutionary forces underlie the observed rapid evolution of the GRR. In contrast, and consistent with the antiviral role of *Drosophila* Ago2, the other domains of this protein display strong evidence of positive selection: they exhibit locally reduced diversity around the gene through selective sweeps, and elevated rates of amino acid substitution (Obbard *et al.* 2006, 2011; Kolaczkowski *et al.* 2011). We have previously argued that this rapid adaptive evolution may be driven by virus-mediated selection, through the action of viral suppressors of RNAi (Obbard *et al.* 2009), such as those seen in *Drosophila* C Virus and *Drosophila* Nora Virus (van Rij *et al.* 2006; van Mierlo *et al.* 2014) . The reportedly high level of variation within the *D. melanogaster* GRR region is therefore surprising, as diversity is expected to be continually removed by nearby selective sweeps. One possible explanation is that the high diversity and differentiation seen in the GRR is purely a result of low constraint on this sequence, combined with high rates of recombination and replication slippage mediated mutations (*e.g.*, Jeffreys *et al.* 1988). Alternatively, if the GRR domains are involved in the antiviral function of Ago2, or interact with viral suppressors of RNAis (VSRs), the high diversity seen in Ago2 GRRs could reflect the action of diversifying selection – which is a common outcome of many models of host−parasite coevolution (*e.g.*, Antonovics and Thrall 1994; Sasaki 2000).

Whether or not the high divergence and diversity seen in GRR2 is an evolutionary consequence of virus-mediated selection, a virus-related role for GRR2 might be reflected by segregating functional variation associated with GRR2 haplotype. In principle, this could be identified by a genome-wide association study (GWAS) such as that which identified *pastrel* (Magwire *et al.* 2012). However, as repeat variants are challenging to reconstruct or identify using short sequencing reads (Treangen and Salzberg 2012), GWAS analyses have largely been limited to SNP and simple structural variation. Thus previous GWAS analyses of viral resistance in *Drosophila* (Magwire *et al.* 2011, 2012) have been unable to test for phenotypes associated with highly repetitive sequences, and instead could only have detected its impact through linkage with neighboring SNPs. But, because the SNP diversity is low in the region surrounding *Ago2*, the scale of linkage disequilibrium (LD) is short in *Drosophila*, and the LD between a SNP and neighboring hyper-mutable loci breaks down rapidly (Sawaya *et al.* 2015), a role for GRR variation in determining viral resistance remains untested.

Here we characterize the sequence diversity of the Ago2 GRR region in insects, and use Pacific -Biosciences SMRT long-read sequencing of RT-PCR amplicons to generate full GRR haplotypes for 127 lines of the *Drosophila* Genetic Reference Panel (DGRP; Mackay *et al.* 2012). We use these data to reexamine the evolution of this domain and its potential role in antiviral defense. In doing so we not only demonstrate the value of long-read technology for performing GWAS when complex repetitive loci are present, but also illustrate the potential challenges associated with such analysis using short-read technology alone. We provide the first robust *Ago2* GRR haplotypes for natural populations, identify likely haplotypes in publicly available short-read data, and quantify differences in the frequency and composition of GRR haplotypes between African and North American populations. Using published GWAS data (Magwire *et al.* 2012) to test for an association between GRR haplotype and virus survival phenotypes, we detect a small but nominally significant association of GRR haplotype with longevity of DCV-infected flies. However, we were unable to confirm this association with a second independent experiment using recombinant inbred lines.

## Materials and Methods

### Comparison of the GRR across insects

We obtained the GRR repeat unit for other insect species by using tBLASTx with default parameters to query all arthropod RefSeq RNA sequences using the *Ago2* region just C-terminal to the GRR from *D. melanogaster*. We manually selected repetitive sequences as input for Tandem Repeat Finder (v4.07b, Benson 1999) with a mismatch and indel penalty of 5 and minimum alignment score of 50. The insect reference tree was inferred using MrBayes (v2.13, Huelsenbeck and Ronquist 2001) with an HKY85 substitution model and γ-distributed rate variation with invariable sites, using conserved sequences from the original tBLASTx search aligned in MUSCLE (v3.8.31, Edgar 2004) as input. The high divergence between GRR sequences, including extensive indel variation, makes it extremely challenging to infer positional homology (*i.e.*, alignment) in the GRR regions (see Figure 2 for *Drosophila* alignments). We therefore used the frequency feature profile phylogeny building tool (v.3.19, Sims *et al.* 2009) to quantify similarity between the GRR of insects, as this approach can be used in the absence of alignment. Frequency feature profiles break the nucleotide or amino acid sequence into a distribution of kmers and compares these distributions against each other taking into account similarity between amino acid residues. The frequency feature profiles were constructed in two ways: in the first, GRR repeat unit consensus sequences were used as input to cluster GRRs, and in the second the entire GRR region was used. In each case, the topology of these clusters were compared to the MrBayes tree, using a kmer size which maximized similarity of the feature frequency profile tree to the MrBayes tree, as it is expected that the GRR shares the same history as the rest of Ago2.

### Sample preparation

We sequenced the GRR region from a subset of the *Drosophila Genetic Reference Panel* (*DGRP*) and seven other closely related *Drosophila* species. The DGRP constitute a collection of highly inbred lines from *D. melanogaster* collected in Raleigh, NC in 2003 (Mackay *et al.* 2012) that have previously been sequenced using the Illumina platform to provide a public resource for GWAS. However, as short-read sequencing cannot easily be used to reconstruct repetitive sequences such as the GRR region of *Ago2*, we generated new amplicon data for the *Ago2* GRR region from 127 of these lines. To avoid sequencing the long intron between GRR1 and GRR2, (RT-)PCR was performed on RNA extracted from 10 flies per line to obtain an amplicon containing the full *Ago2* GRR1 and GRR2 regions. For *Drosophila* species other than *D. melanogaster*, sample origins are as described in Longdon *et al.* (2011). For all species, RNA was extracted using Trizol (Ambion) according to the manufacturer’s instructions. Three forward primers were designed separately for the *Drosophila melanogaster/simulans/mauritiana* clade, the *Drosophila yakuba/erecta/santomea* clade, and for *D. ananassae* based on published genome sequences (PCR primer sequences: 15F *D. yakuba*: ATGGGAAAGAAGAACAAATTCAAGG; 30F *D. melanogaster*: GAACAAGAAAGGAGGACAGG; ^18^F *D. ananassae*: ATATAAGGATGACGGGAAGC). PCRs shared a single reverse primer designed to amplify all species (1550R CAGCTTATCCACCGAGTAGCA) except for *D. ananassae* (GTCGACATTAAGAAACGGTT). Paired barcode sequences from the Pacific Biosciences SMRT Portal v1.4 were added to the 5′ end of each primer, along with the padding sequence GGTAG. Barcoded amplicons were then combined into 10 pools of 16 samples and gel purified for sequencing.

### Long-read amplicon sequencing and analyses

**Figure 3:**
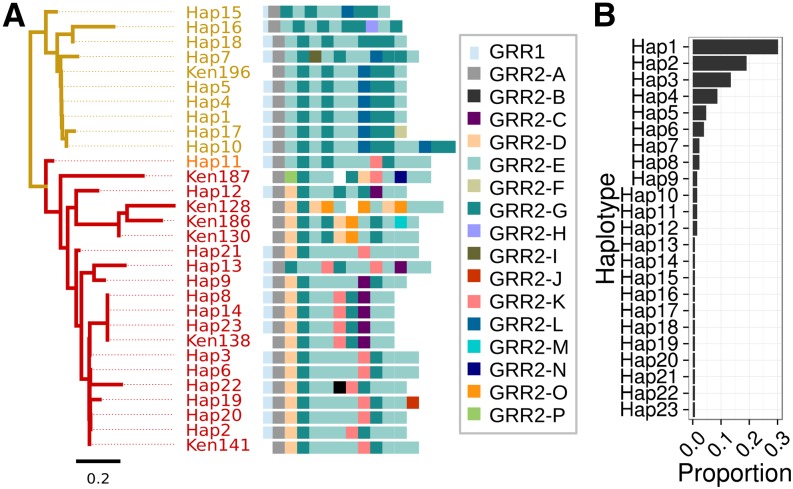
Variation in the GRR repeat sequence and structure. (**A**) Similarity clustering analysis of GRR2 haplotypes reveals two large groups of *D. melanogaster* haplotypes (gold and red) and one putatively recombinant haplotype (orange). Haplotypes are illustrated using color-codes for the 16 distinct repeat units corresponding to the arbitrary character identifiers A–P. In some Sanger-sequenced Kenyan haplotypes (labeled ‘Ken’) a repeat unit could not be determined, denoted by a white square. Note that repeat unit L is diagnostic of haplotype group α, and units D and K are diagnostic of group β. (B) Histogram of the frequency of each haplotype in the DGRP population. Most haplotypes occur at low frequency, with some high and intermediate frequency haplotypes.

Samples were pooled in groups of 16 and subject to Pacific Biosciences SMRT-cell sequencing (NERC Biomolecular Analysis Facility, Liverpool). *D. melanogaster* raw reads were demultiplexed and filtered in the SMRT portal by five minimum passes around the circular template, requiring a minimum predicted accuracy of 70%, a minimum insert size of 1000 bases, and a minimum barcode score of 22. From these, five-pass circular consensus sequences (5CCS) were called for each read (raw read processing was performed by NERC Biomolecular Analysis Facility, Liverpool). Although these 5CCS reads may still contain errors, to obtain the final consensus sequence for each fly line we grouped all 5CCS reads by length, and then removed reads whose length was observed in <10 reads. This filtering resulted in a single peak of read lengths for each amplicon (*e.g.*, Supplemental Material, Figure S1) in all but one fly line. In this one line (DGRP-306), we detected two high-frequency haplotypes, suggesting that this line is heterozygous at the GRR region, and this sample was excluded from all subsequent analyses. Consensus sequences from 5CCS reads within the length class resulted in high-confidence haplotypes from 127 of the DGRP lines, which were used in further analyses. In addition, eight haplotypes from a Kenyan (Nairobi) population, which were previously obtained by Sanger sequencing of long PCR products (Obbard *et al.* 2006), were also included in the analysis. GRR sequences were also obtained from single lines of *D. simulans*, *D. sechellia*, *D. yakuba*, *D. santomea*, *D. erecta*, and *D. ananassae*. These were analyzed in the SMRTportal with the parameters described above, but with a minimum insert size of 500 bp. We used BLAST (2.2.31+, Camacho *et al.* 2009) to recover the species of each long 5CCS read from the coding sequence to the 3′ of GRR2, then we grouped reads by species and read length. Peaks in read length were again assumed to be indicative of a distinct amplicon, and analyses were performed as in the *D. melanogaster* samples. To cluster haplotypes (Figure 3) by repeat unit, the distinct repeat units observed in *D. melanogaster* were each labeled with an identifying letter, such that a haplotype can be denoted a string of repeat unit identifier letters. We then used text-based feature frequency profiles (hash length of 2) to cluster and visualize haplotypes by repeat unit similarity (Sims *et al.* 2009).

### Characterization of GRR repeats in published short-read data

To explore the utility of published short-read sequencing in the reconstruction of the *Ago2* GRR, we obtained short-read sequences of DGRP (Accession number: PRJNA36679, Mackay *et al.* 2012) and *Drosophila* ‘Nexus’ lines (Lack *et al.* 2015: Table S1 Accession numbers). To retain reads deriving from the region of interest, all reads were mapped to our full set of 127 sequenced GRR haplotypes using Bowtie2 (v. 2.2.4, Langmead and Salzberg 2012) with default parameters, retaining all read pairs for which at least one mate mapped. An attempt was made to assemble these reads *de novo* using Velvet (v1.2.10, Zerbino and Birney 2008), using the hash length for each individual that maximized contig length, and using the expected coverage and insert length data provided by the sequence read archive.

To assess whether the distribution of repeat units in short-read sequences could be used to infer GRR2 haplotypes, we used Jellyfish (v.2.2.3, Marçais and Kingsford 2011) with a kmer size of 69 (the size of a GRR repeat in *D. melanogaster)* and a lower coverage bound of 2 (although this parameter had no qualitative effects when varied from 0 to 10) to infer counts for known repeat units in each sample. To ensure we only included samples with sufficient coverage of the GRR to reliably infer haplotypes, we filtered out those samples without reads supporting repeat unit GRR2-G and repeat unit GRR2-A, and without ten reads supporting GRR2-E (these repeat units were shown to occur in all 127 DGRP samples using PacBio amplicon sequencing, with GRR2-E being most common). The retained samples were then normalized by total read count to obtain a proxy for relative abundance of repeat units in each sample.

### Linkage disequilibrium analysis

We combined our GRR haplotype data with known SNPs and indels within 5 kb on either side of *Ago2* from the DGRP dataset (http://dgrp2.gnets.ncsu.edu/data/website/dgrp2.tgeno), replacing any reported sequence within the GRR with our own long-read sequence data. We then calculated a multiallelic extension of *r*^2^ (Hill and Robertson 1968), which provides an accurate metric of LD among multiallelic loci (Zhao *et al.* 2007). The analysis was performed using our data coded either as entire haplotypes (and therefore highly multiallelic), or as a series of SNPs and indels from alignment of the haplotypes.

The rapid increase in frequency of a beneficial allele is expected to lead to extended regions of high LD around the swept allele (termed ‘haplotype homozygosity’; Sabeti *et al.* 2002) and to quantify this we used the program nSL (v.0.47, Ferrer-Admetlla *et al.* 2014) to calculate the nSL statistic for the regions surrounding the GRR. The nSL statistic is similar to the more widely used iHS statistic (Voight *et al.* 2006), except that distance is measured as the number of segregating sites rather than map distance, making it more robust to recombination rate variation. Moving along the sequence, at each polymorphic site nSL calculates the average number of consecutive polymorphisms associated with either the ancestral or derived allele in question. Either exceptionally large or small values of the nSL statistic are evidence that a variant has rapidly increased in frequency. For *D. melanogaster* we polarized the sites with the *D. simulans* genome by parsimony, aligned by LastZ (v.1.02.00), and standardized the nSL statistic by allele frequency.

### Association with viral phenotypes and infections

To test whether variation in the GRR haplotype is associated with variation in viral resistance, we used data from previous GWAS studies (Magwire *et al.* 2011, 2012) of the DGRP lines for resistance against three different viruses. These were *Drosophila* C virus (DCV, a horizontally transmitted and highly pathogenic Dicistrovirus naturally infecting *D. melanogaster* (Brun and Plus 1980; Webster *et al.* 2015); *D. melanogaster* σ virus (DMelSV: a vertically transmitted Rhabdovirus naturally infecting *Dmel*; Brun and Plus 1980; Longdon *et al.* 2012; Webster *et al.* 2015), and Flock House virus (FHV, a horizontally transmitted Alphanodavirus naturally infecting beetles, closely related to Newington virus of *D. immigrans* (Webster *et al.* 2016). We fitted general linear mixed models using the R package MCMCglmm (v2.22, Hadfield 2010) with DGRP line and replicate block (block equivalent to date for FHV and DCV) as random effects, and known segregating functional variants (*pastrel* for *DCV*, and *ref(2)p*, *CHKov*, and *ge1* for DMelSV) and GRR haplotypes as fixed effects.

The final model was:

$$Y_{\mathrm{ijkl}}∼\mu +\mathrm{pastre}l_{i}+\mathrm{haplotyp}e_{j}+\mathrm{lin}e_{k}+\mathrm{bloc}k_{l}+\hat{u}$$

Where μ is the mean survival time and Ɛ is a normally distributed error term. If LD is sufficiently large, it may be difficult to separate the effect of GRR haplotype from the effect of (partially) linked SNPs. Therefore, to examine whether the GRR haplotype is acting as a marker for a neighboring causal SNP, we also fitted models in which each flanking SNP was tested for an association with mortality, and then selected those which were nominally significant (with no correction for multiple testing) for inclusion in the model outlined above, to verify any observed effect was due to the GRR.

### Recombinant inbred line infections

To further test for an association between *Ago2* GRR haplotype and viral resistance, we experimentally infected recombinant inbred lines from the *Drosophila* Synthetic Population Resource (DSPR) (King *et al.* 2012) with DCV. We categorized lines by *Ago2* GRR haplotype groups based on presence of reads containing the repeat units GRR2-L (as a marker for haplotype group α) or GRR2-D and GRR2-K (as markers for haplotype group β) in the short-read data for the DSPR parental lines. The length of the linked region around the GRR region was calculated in each recombinant inbred line, and 100 lines from each haplotype group were selected with the aim of minimizing the impact of linked variants (*i.e.*, lines were chosen on the basis of nearby break points). Infections were performed by injecting DCV abdominally into 10 flies per vial with an average of three vials per line, at 10^5^ TCID50, chosen on the basis that this dosage caused mortality in ∼1 wk. Flies were kept at 25° in agar vials and monitored for 7 d postinfection (DPI) with mortality recorded on each day. The data were analyzed using a binomial regression in MCMCglmm with the model:

$$Y_{\mathrm{ijklm}}∼\mu +\mathrm{DPI}+\mathrm{DP}I^{2}+\mathrm{pastre}l_{i}+\mathrm{GRRhaplotyp}e_{j}+\mathrm{lin}e_{k}+\mathrm{Via}l_{l}+\mathrm{Dat}e_{m}+\mathrm{Date : DPI}+\mathrm{Vial : DPI}+\varepsilon$$

We followed Longdon *et al.* (2011) in coding mortality (Y) as a number of ‘successes’ (the number of flies remaining alive in a vial on a certain day) and ‘failures’ (the number of flies that died on a certain day). This model fits GRR genotype, *pastrel* parent of origin (as a proxy for *pastrel* genotype), and DPI as fixed effects. DPI is encoded as both a linear and quadratic predictor, as mortality tends to decrease after the peak infection. We included DSPR line (genetic background), vial, and date as random effects, allowing for interactions between the DPI and either date or vial effects.

### Data availability

Haplotype sequences have been submitted to GenBank under the accession numbers KX069093 - KX069218.

## Results

### Evolution of the GRR across insects

The presence of a GRR region in *Ago2* is conserved across the arthropods, but the GRR evolves extremely rapidly, and the diverse structure of the GRR makes alignment and assembly of these regions challenging. Some species have multiple repeat units, such as *Megachile rotundata* (leafcutter bee) – with repeat units QRRSLAPHG and LKQQQQPLAPQQHHTFA – others have nested repeat units, as in *Tribolium castaneum* (flour beetle), where a region with multiple repeats with consensus QQQWQQQQPQPHP appears to have been duplicated. To circumvent the challenge of alignment difficulties, feature frequency profiles (distance matrices produced by comparing the distribution of kmers across different sequence) of the GRR and amino acid composition were used to quantify similarity without alignment. Conservation of either amino acid composition or repeat unit sequence could imply functional significance of the GRR, and so we examined the GRR of 34 insect species (Figure 1). Trees from feature frequency profiles were constructed from the entire GRR (Figure 1B) or from the consensus repeat unit (Figure S2), and compared to the Ago2 gene tree (Figure 1A and Figure S3). In both cases, the GRR region sequences clustered broadly according to known species relationships but do not reliably reflect more divergent evolutionary relationships. For example, the relationships between *D. melanogaster*, *D. simulans*, *D. erecta*, and *D. yakuba* were correctly resolved, but the Drosophilidae did not cluster together in any distance measure (for alignments, see Figure 2). This divergence is in part due to structural differences between GRRs (Figure 1C), as the number and size of repeat units is variable, even between closely related species. In addition, trees made from repeat unit consensus sequences are unable to correctly cluster hymenopterans and dipterans, indicating that the divergence is unlikely to be due to assembly artifacts. Alternatively, amino acid sequence composition is similar across the species analyzed, with glutamine the most frequent amino acid residue in all species analyzed except *Athalia rosae* (turnip sawfly; Figure 1). This conservation is further illustrated throughout the Drosophilidae (and closest outgroup *M. domestica*), whose GRR is strikingly glycine-rich. These observations argue that although the GRR sequence and structure evolves quickly, the composition may be under selective constraint, implying functionality.

### Haplotypes and repeat units in D. melanogaster Ago2 GRR

**Figure 4:**
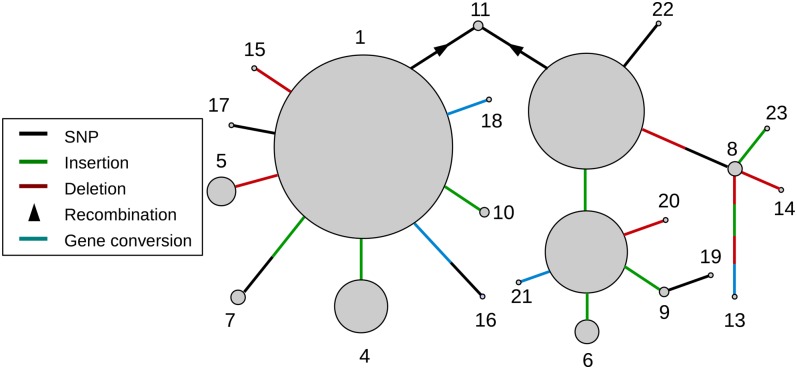
Reconstructed recent history of the GRR. A network showing the inferred relationship between different GRR haplotypes (circles), with circle area corresponding to the frequency in our sample of the DGRP, and connectors representing different mutation or recombination events. Note that there are some haplotypes whose relationship is not easily linked with the others, for example, *GRR Hap13* is unlike any other haplotype sequence and there are large differences between *GRR Hap1* and *GRR Hap2*. In other cases it is not clear whether convergent mutation or recombination produced a particular haplotype, for example, the different GRR1 variants each occur in the background of multiple GRR2 variants (see Figure 2).

We found extensive repeat polymorphism among the DGRP lines. Among the 127 lines sequenced, we identified three different GRR1 haplotypes and 18 GRR2 haplotypes, between which there is no detectable LD (Figure S4). GRR1 and GRR2 regions could be identified in other *Drosophila* species we sequenced as well, however the repeat unit sequences differ, as described above (Figure 2). All GRR1 haplotypes comprise one to three perfect repeats of the sequence PQQLQQ, with two repeats being most common (Figure 3). The GRR2 is more complex, with 12 different repeat units (labeled GRR2-A to GRR2-L, Figure 3). The distinct repeat units seen in *D. melanogaster* are all within three nucleotide differences of each other and a consensus sequence of GQQQGGHQQGRQGQEGGYQQRPP (Figure 2), and occur 10–15 times in tandem. Most of the GRR2 sequence is composed of two repeat units: GRR2-E (occurring 4–8 times per haplotype) and GRR2-G (occurring 1–6 times per haplotype), which differ at a single amino acid position. In contrast, the majority of repeat units are rare, only occurring in one haplotype, and are most likely the result of recent single base-pair mutations (*e.g.*, GRR2-J). Together, the GRR1 and GRR2 alleles form 23 distinct GRR haplotypes in our dataset. Clustering GRR haplotypes by repeat unit composition (see *Materials and Methods*) identifies two largely distinct haplotypes classes (colored gold and red in Figure 3), and one putatively recombinant haplotype (*GRR Hap11* – colored orange) in the DGRP sample. Based on this clustering dendrogram, we have attempted to reconstruct the recent history of the GRR region, as most haplotypes appear to differ from one another by one or two mutation or recombination events (single base changes, whole-repeat insertions and/or deletions, and gene conversion) (Figure 4).

Many of these GRR haplotypes occur at a low frequency in the DGRP, with 11 of the 23 haplotypes occurring only once in our sample (Figure 3 and Figure 4). There are three high-frequency haplotypes (*Hap1*, *Hap 2*, and *Hap3*) with the latter two differing by only one repeat unit. Interestingly, there are many differences between the *Hap1* and *Hap2/Hap3* groups (hereafter referred to as haplotype groups α and β), such that no simple single mutational event could convert one to the other. Further, the haplotypes in haplotype group α occur at low frequencies and are no more than two mutational events from *Hap1* itself, suggesting they may have been formed recently. This observation is at odds with the high frequency of *Hap1*, and may indicate a recent increase in the frequency of the GRR α group. In support of this idea, despite the approximately equal frequency of individuals in haplotype groups α and β, nucleotide diversity in Ago2 and a 100 kb surrounding region is much lower in haplotypes from the α clade than those in the β clade, indicating this *GRR Hap1* is younger than expected given its relative frequency (Figure S5). Nevertheless, there does not seem to be any evidence for significant extended haplotype homozygosity in the remainder of the gene (Figure S6).

We also analyzed eight Sanger-sequenced GRR2 haplotypes from a Kenyan population of *D. melanogaster* (Obbard *et al.* 2006) and compared them to the DGRP haplotypes (Figure 3). Notably, seven of the eight Kenyan haplotypes were distinct from each other, and in these seven haplotypes, four new repeat units were found (GRR2-M, GRR2-N, GRR2-O, GRR2-P; Figure 3). This may suggest that the diversity in the DGRP is a subset of African diversity, as expected from the evolutionary history of this species (Lachaise and Silvain 2004; reviewed in Stephan and Li 2007). GRR2-L, the defining repeat unit of the GRR α clade found in the DGRP (gold branches in Figure 3), was rare in the sample of eight Kenyan sequences, although not absent, suggesting the presence of substantial population structure in GRR.

**Figure 5:**
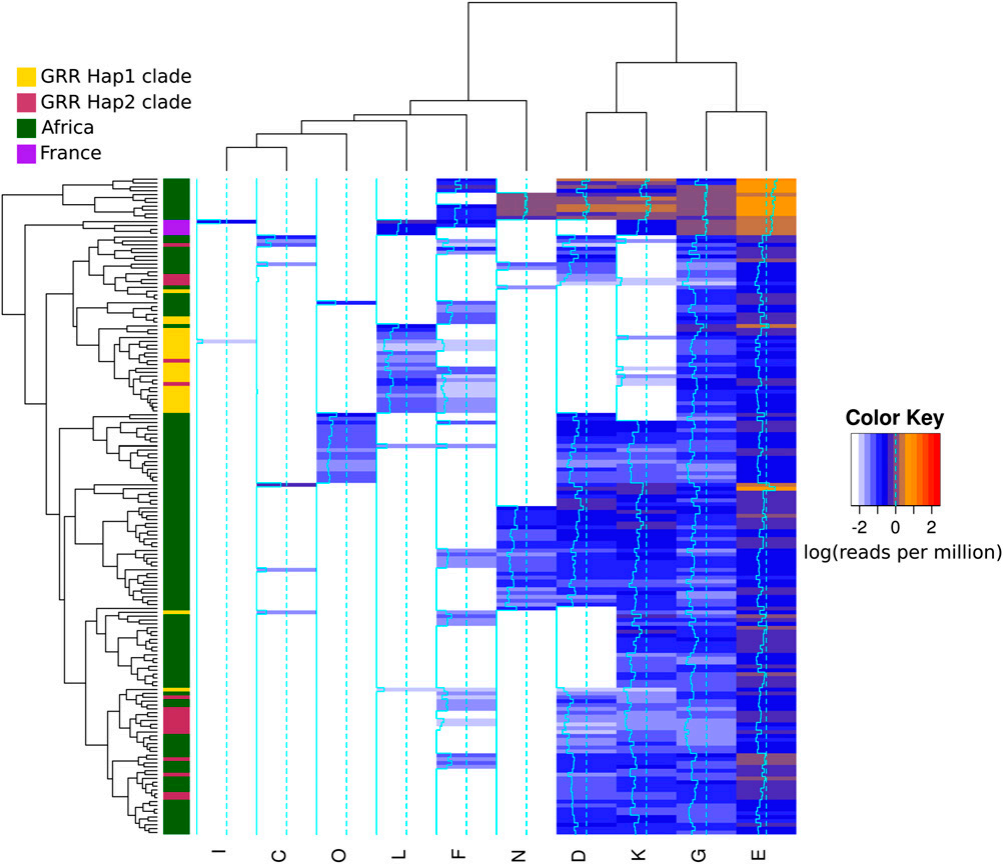
Repeat units in the *Drosophila* Nexus lines. Clustering of the distribution of repeat units in short-read data for a sample in *Drosophila* populations taken from the nexus dataset (Lack *et al.* 2015). Lines were excluded if no short reads were found for ubiquitous repeat units (see *Materials and Methods*). *GRR* α clade and *GRR* β clade are those found in the clustering analysis of Figure 2. DGRP *GRR* α clade appears to be derived from an ancestral African population, whereas *GRR* β clade is more divergent, and represents a subset of African diversity. Also, notice the existence of population-specific repeat units (*e.g.*, repeat units *O* and *N*) and population-specific co-occurrence of repeats (*e.g.*, repeat units *L* and *K* in France).

Although we were unable to reconstruct reliable GRR haplotypes from short-read data, we were able to identify the presence of specific repeat units such as *GRR-L*, which in the DGRP is diagnostic of the *GRR* α clade, and GRR-D and K, which are diagnostic of *GRR* β clade. We therefore took advantage of the recent release of the *Drosophila* Genome Nexus, which includes the DGRP as well as individuals sequenced from Africa and France (Lack *et al.* 2015), and characterized the distribution of repeat units in these lines (Figure 5). There are repeat units specific to both African (*GRR2-O* and *GRR2-N*) and North American (*GRR2-B*, *GRR2-H*, *GRR2-I*, and *GRR2-J*) populations, although those peculiar to North America were all rare variants. However, French lines also cluster together, characterized by co-occurrence of *GRR2-L* and *GRR2-K* – the defining features of each of the two large classes defined by the DGRP – indicating these lines may be recombinants or heterozygotes. Short-read data also suggested that *GRR2-L* is rare in Africa, whereas *GRR-D/K* are common and often co-occur. These observations indicate that the *GRR* α clade has risen in frequency since *D. melanogaster* arrived in North America.

### Associations between GRR haplotypes and survival during virus infection

The role of the GRR region is unknown, but as Ago2 is a major effector of antiviral immunity in *Drosophila* (van Rij *et al.* 2006), it could function during antiviral defense. Using previously published survival data, we found no significant association between GRR haplotype and resistance to FHV [95% CI for GRR effect: (−0.12, 0.4), *MCMCp* = 0.30] or σ virus [95% CI for GRR effect: (−0.15, 0.03), *MCMCp* = 0.242] infection in the DGRP. However, when fitting GRR haplotype as a fixed effect, we found that *Hap3* alleles increased longevity following challenge with DCV by ∼0.7 d relative to *Hap1* alleles [*MCMCp = 0.012*, (95% CI 0.21 to 1.17) d; Figure S7]. This appears to be due to the GRR2 region, as inclusion or exclusion of GRR1 state had no effect. A second model in which GRR1 data were excluded, identified both *Hap2* and *Hap3* as significantly increasing survival relative to Hap1 [*Hap2: MCMCp* = 0.006, 0.56 (0.15 to 0.97) d]; [Hap3: *MCMCp* = 0.006, 0.64 (0.23 to 1.07) d] . However, the observed effect is small relative to the effect of the known resistance variant pastrel^T^ (Magwire *et al.* 2012), which increases longevity in the same experiment by 2.07 d [MCMCp < 0.001, (1.58 to 2.54)].

Given the small size of the effect, multiple tests across viruses, and marginal *P*-values, we elected to perform a second independent test using a subset of the recombinant inbred lines provided by the DSPR (King *et al.* 2012). In this experiment, although mean survival time was slightly greater for haplotype group β than group α, this trend was not significant (pMCMC = 0.646; Figure S7). The same was true if parent of origin was used as a fixed effect instead of GRR genotype. This is unlikely to be due simply to low power, as we were able to detect a significant association with genotype at the (albeit larger effect) resistance locus *pastrel* (pMCMC < 0.001). We are therefore unable to replicate the nominally significant effect of GRR haplotype on survival in the DGRP.

## Discussion

### GRR amino acid composition is conserved, but repeat unit sequence and structure is not

We observe a high degree of sequence divergence in the Ago2 GRR across insect species. Even over very short timescales, there is high divergence in copy number and repeat unit sequence (Figure 1). This could be explained by a high rate of partial interrepeat replication slippage, causing the creation of new repeat units from the existing ones, and making the sequence unrecognizable in a relatively short period of time (*e.g.*, Dmel and Dsim GRR2 sequences, which are highly divergent despite only 2.5 My since they shared a common ancestor). In contrast to the sequence of the GRR, we find that the amino acid composition is conserved across the insect species analyzed. Although mutational processes can drive sequence conservation in microsatellites, the required concurrent conservation of reading frame in the GRR makes this possibility unlikely. Based on these observations, we envision a scenario where stabilizing selection acts at the level of amino acids (*e.g.*, to maintain a certain charge or hydrophobicity) but either lack of constraint or positive selection acts at the level of repeat unit sequence and structure.

### GRR repeats are highly variable within D. melanogaster, and may be under selection

Repeat number polymorphism in the Ago2 GRR of laboratory lines was previously reported by Hain *et al.* (2010), and our long-read sequencing of a natural population of *D. melanogaster* (the DGRP; Mackay *et al.* 2012) confirms that this variation is also widespread in the wild. However, our sequencing demonstrates considerable length convergence among haplotypes, such that only seven different haplotype lengths were present among the 23 distinct haplotypes, and 8 of the 23 distinct haplotypes had the same length (1.035 kb; Figure 3) – including haplotypes in both the α and β GRR groups. We found that the haplotypes falling into the α clade appear to have recently increased in frequency in the North American (DGRP) population. This is supported by a lower diversity surrounding *GRR* α than *GRR* β clade haplotypes, despite the expectation that neutral diversity in linked regions should scale positively with the frequency of the allele. The increase in frequency of the *GRR* α clade could be due to drift (*e.g.*, during a bottleneck) or selection, such as parasite-mediated selection acting on *Ago2* GRR repeat region itself. However, given the known selective history of *Ago2* (Obbard *et al.* 2011), this distribution of haplotype frequencies could also be explained by incomplete linkage to a nearby hard sweep carrying GRR Hap1 to a high frequency (*e.g.*, Schrider *et al.* 2015).

### Ago2 GRR variation is not strongly associated with survival after viral challenge

In other genes, extended low-complexity tracks of a single amino acid have known functions, including having been implicated in transcription factor binding (*e.g.*, glutamine, proline, alanine), protein aggregation (glutamine), and cellular localization (histadine), and recently the Q-rich opa repeats of *Notch* have been found to be involved in developmental defects (Gerber *et al.* 1994; Salichs *et al.* 2009; Gemayel *et al.* 2015; Rice *et al.* 2015). But, although the long-term conservation of the Ago2 GRR among pancrustacea argues that it is maintained by selection, the function of this repeat region remains unclear. As VSRs have been proposed as the likely drivers of the rapid protein evolution of Ago2 (Obbard *et al.* 2009), and high diversity is predicted by many models of host–parasite coevolution (*e.g*., Antonovics and Thrall 1994; Sasaki 2000) it is tempting to speculate that the Ago2 GRR may play a role in VSR evasion. For example, the GRR could act to cover residues that underlie Ago2-VSR interactions, or as a bait region, sequestering VSRs away from the catalytic residues of Ago2. However, although *Ago2* GRR showed a significant association with survival after DCV infection in our reanalysis of published data from 127 of the DGRP lines, we were unable to replicate this using selected lines from the DSPR. These conflicting results may reflect a false positive from the DGRP analysis, or low power in the DSPR analysis, perhaps due to the challenge inherent in categorizing GRR haplotypes using short-read data. However, in either case, it is clear any association must be weak relative to previously identified segregating functional polymorphisms, such as *pastrel*.

### The potential importance of complex repeat sequences in GWAS studies

We find that LD within the GRR, and between the GRR and surrounding variants, is low (Figure S4), indicating that any phenotypic association with this repeat region would be difficult to identify through GWAS using linked sites only. Additionally, the convergence in length between highly divergent GRR haplotypes means that simple length assays may not be suitable to differentiate between haplotypes and may miss important variants. More generally, our study suggests that short-read sequencing, such as that currently employed by the majority of association studies, is not a viable option for repetitive regions, as we were only able to assemble one correct *Ago2* GRR haplotype among the 117 DGRP datasets using public sequence read data. Clustering by repeat unit presence in short-read data confirms our PacBio-sequenced haplotypes (Figure 5), but may only be useful if there is prior knowledge to the possible repeat units in a population and if the region is sequenced in high depth. For example, reads with repeat units *GRR2*-A, *GRR2-G*, and *GRR2-E* (which occur in every haplotype) were not always detectable in the short-read data for a sample. This indicates that GRR coverage can be low and that incorrect haplotype inference was not only due to assembly errors, but also may indicate that the GRR region has unusually low coverage – perhaps because it is not conducive to short-read sequencing. Together, these attributes argue that sequencing repetitive regions can provide a depth of understanding not attainable by looking at length variation alone.

## Supplementary Material

Supplemental Material
