# Supplementary material for: Variation and Evolution in the Glutamine-Rich Repeat Region of Drosophila Argonaute-2
Source: G3 (Bethesda). 2016 Jun 16;6(8):2563–72. doi: 10.1534/g3.116.031880 (PMC4978909; doi:10.1534/g3.116.031880)
Supplement: Supplemental Material [file supp_6_8_2563__index.html]

Variation and Evolution in the Glutamine-Rich Repeat Region of Drosophila Argonaute-2 — Supplemental Material 

# Variation and Evolution in the Glutamine-Rich Repeat Region of *Drosophila* Argonaute-2

## Supplemental Material for Palmer and Obbard, 2016

**Files in this Data Supplement:**

- Figure S1 - PacBio sequencing reads of the GRR in the DGRP. (.pdf, 23 KB)
- Figure S2 - FFP profile clustering from repeat unit consensus sequence. (.pdf, 135 KB)
- Figure S3 - FFP profile clustering from conserved Ago2 sequence. (.pdf, 125 KB)
- Figure S4 - Linkage between GRR and surrounding 10KB and r2 values between haplotypes and single GRR SNPs. (.pdf, 396 KB)
- Figure S5 - Lower diversity in the GRR alpha group relative to GRR beta group. (.pdf, 62 KB)
- Figure S6 - AGO2 nSL signature in the DGRP and haplotype bifurcation diagram. (.pdf, 1.6 MB)
- Figure S7 - Involvement of GRR during DCV infection. (.pdf, 51 KB)
- File S1 - GRR2 Fasta file containing the sequences of each annotated repeat unit. (.fasta, 2 KB)
