# Supplementary figures and images for: Variation and Evolution in the Glutamine-Rich Repeat Region of Drosophila Argonaute-2
Source: G3 (Bethesda). 2016 Jun 16;6(8):2563–72. doi: 10.1534/g3.116.031880 (PMC4978909; doi:10.1534/g3.116.031880)

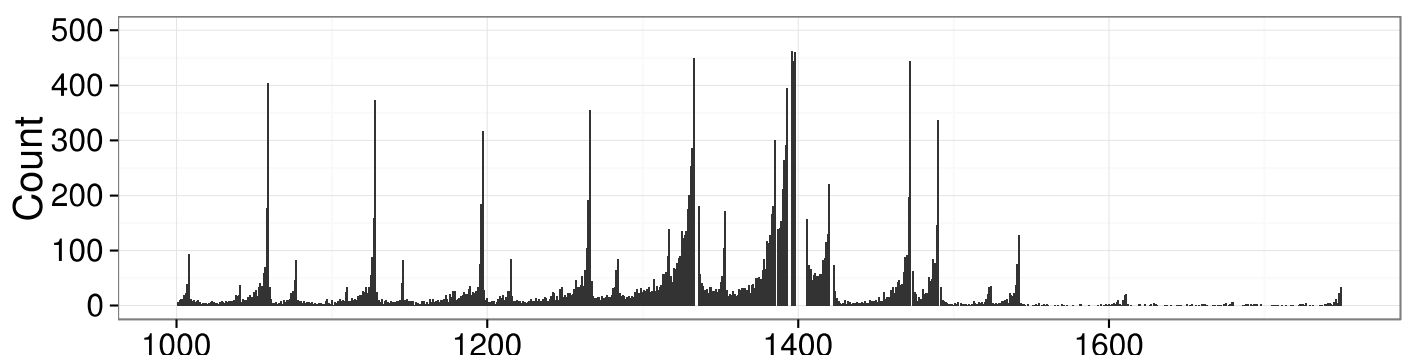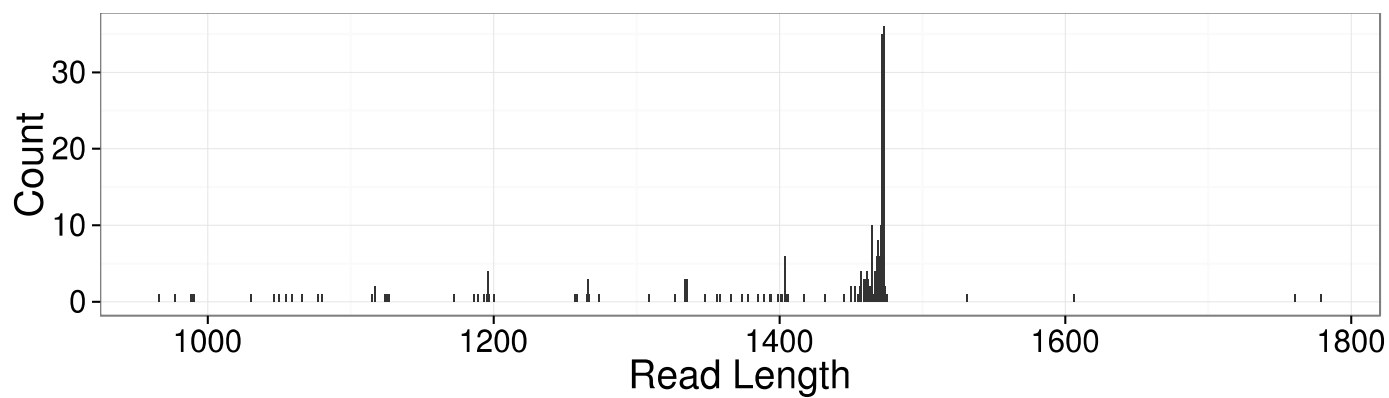

Supplement: Supplemental Material [file supp_g3.116.031880_FigureS1.pdf]

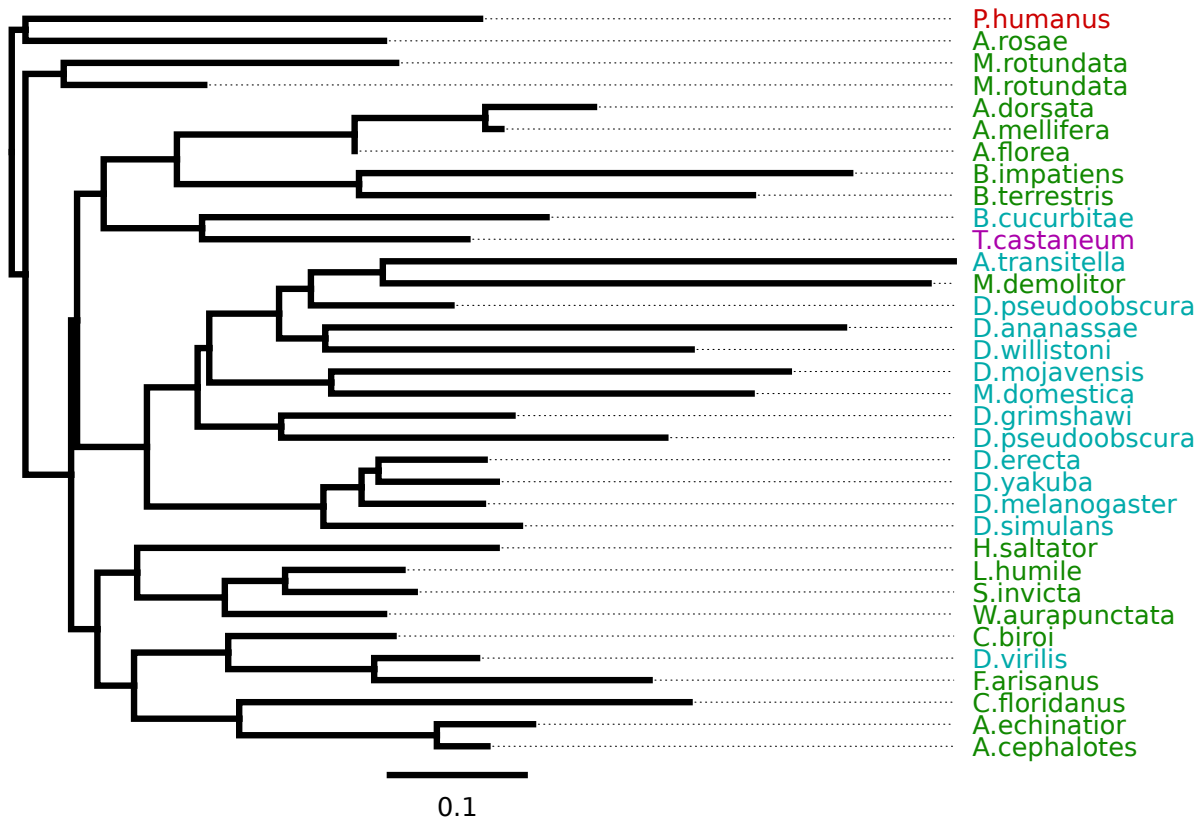

Supplement: Supplemental Material [file supp_g3.116.031880_FigureS2.pdf]

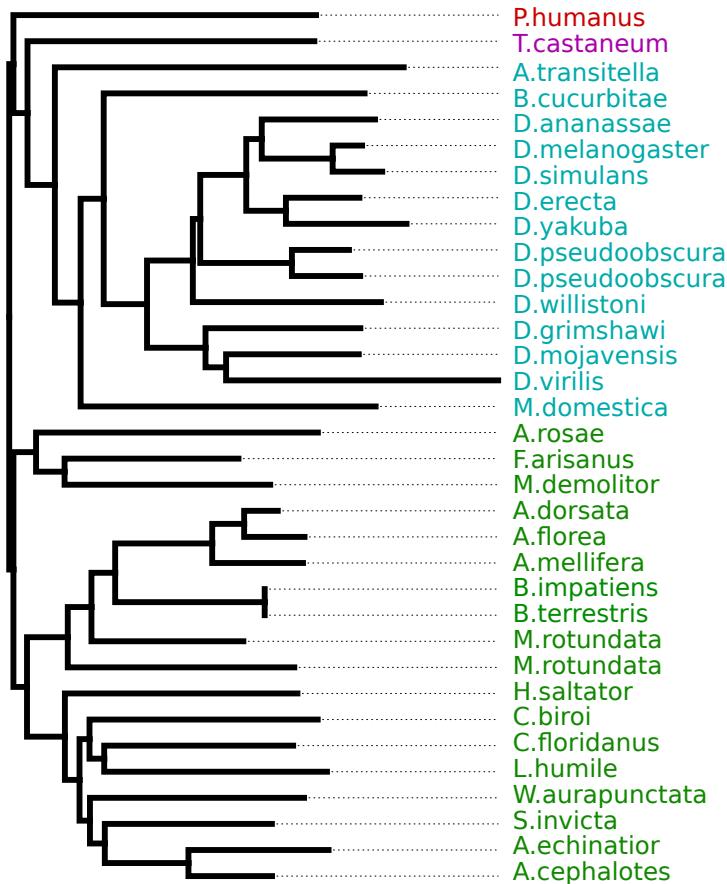

0.09

Supplement: Supplemental Material [file supp_g3.116.031880_FigureS3.pdf]

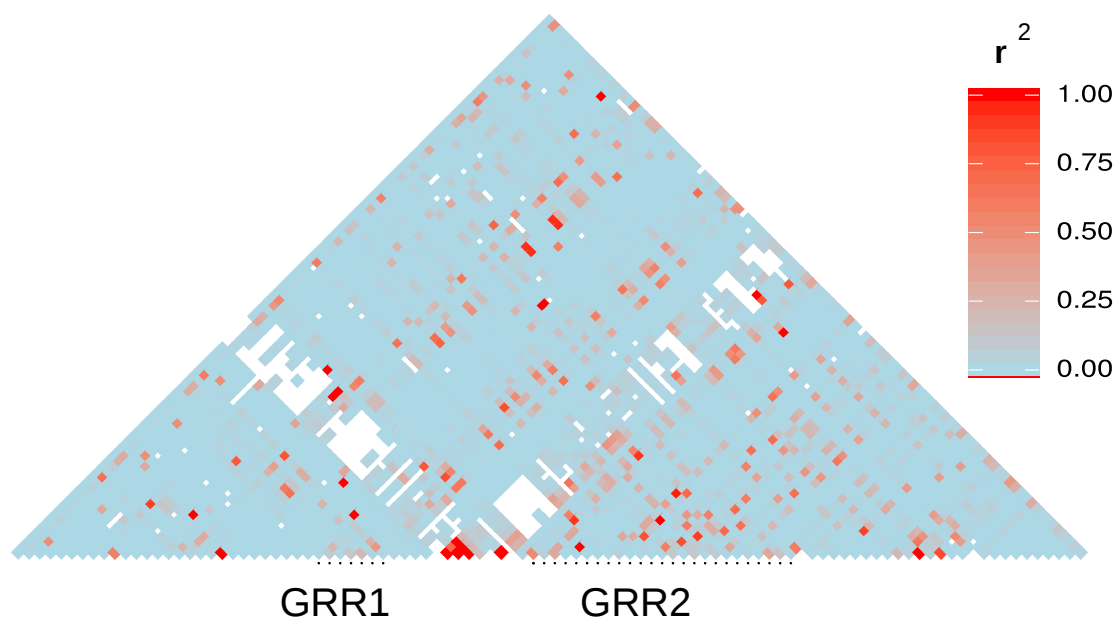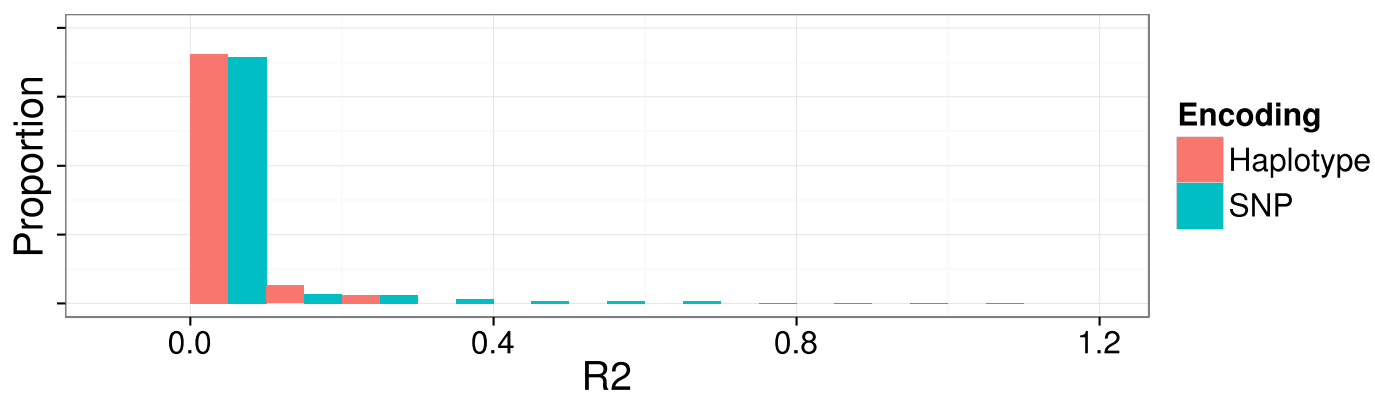

Supplement: Supplemental Material [file supp_g3.116.031880_FigureS4.pdf]

$\log(\text{GRR alpha diversity}/\text{GRR beta diversity})$

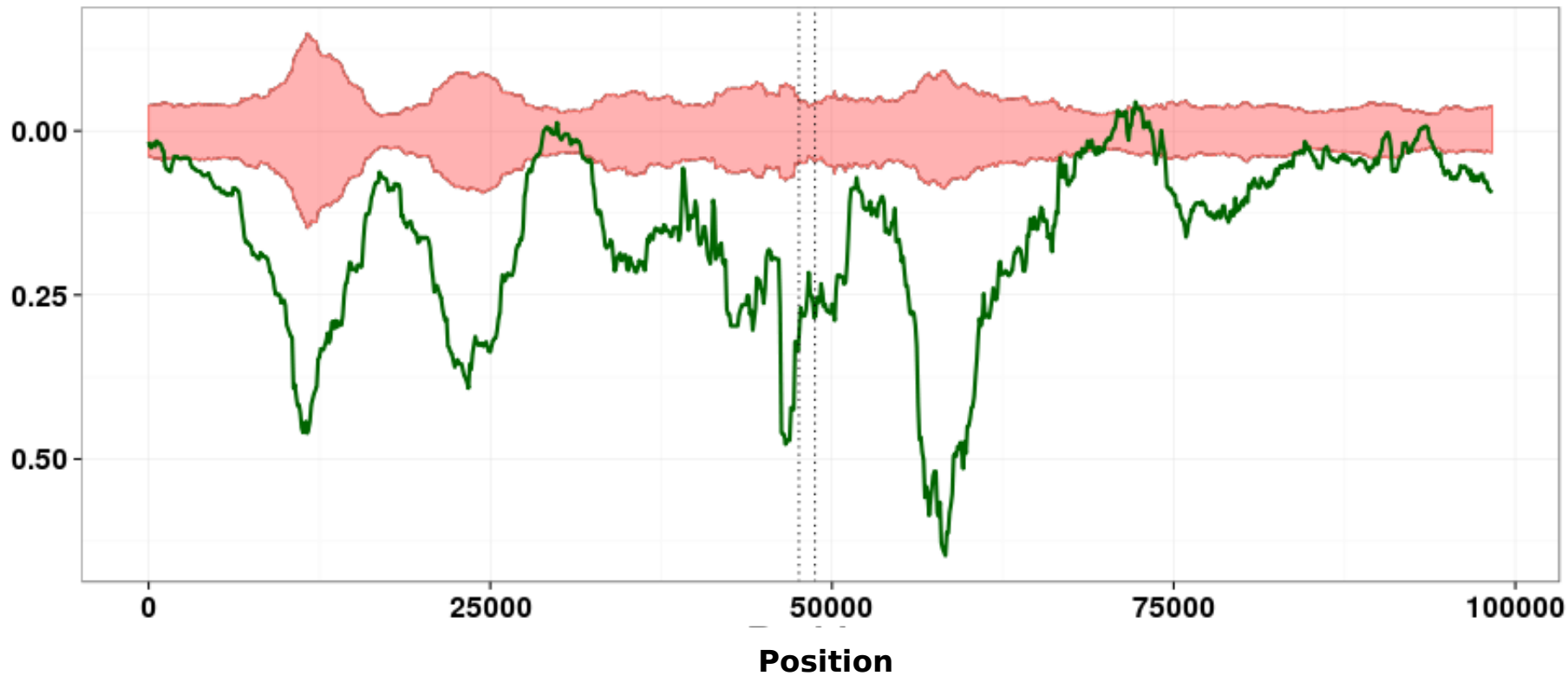

Supplement: Supplemental Material [file supp_g3.116.031880_FigureS5.pdf]

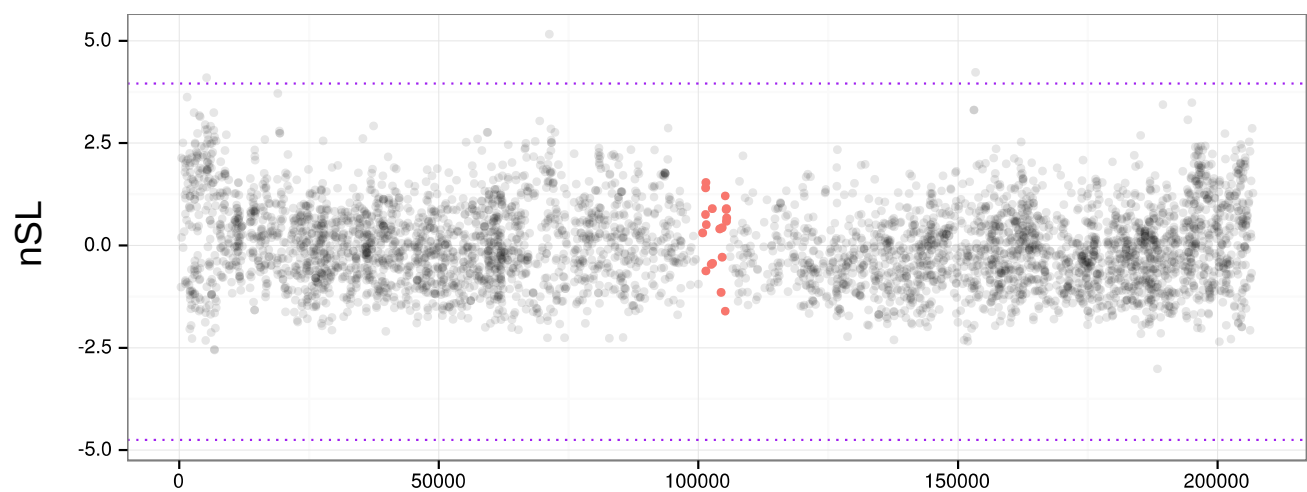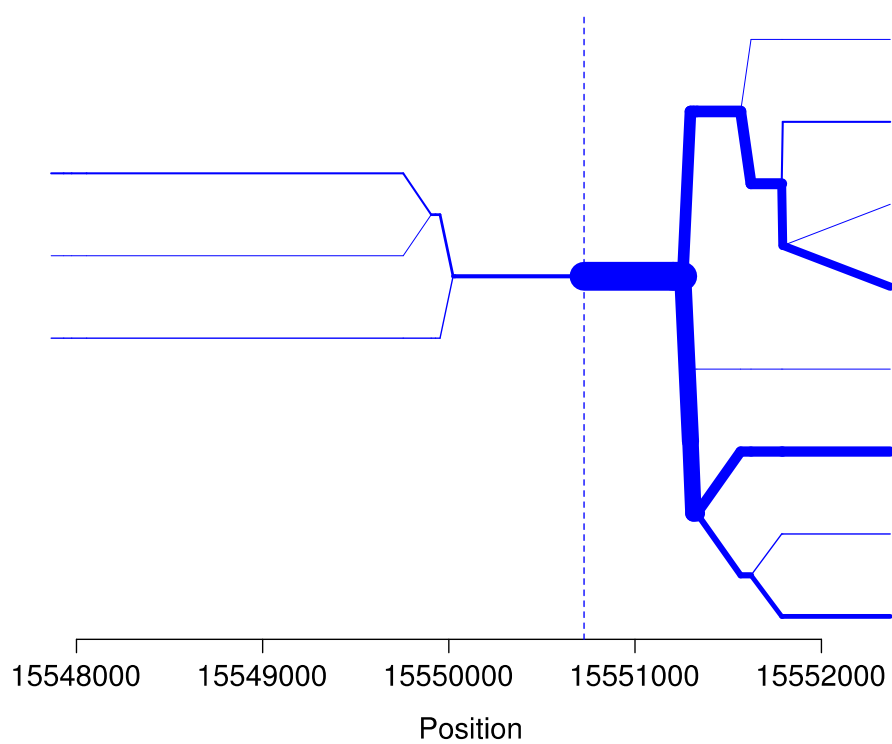

Supplement: Supplemental Material [file supp_g3.116.031880_FigureS6.pdf]

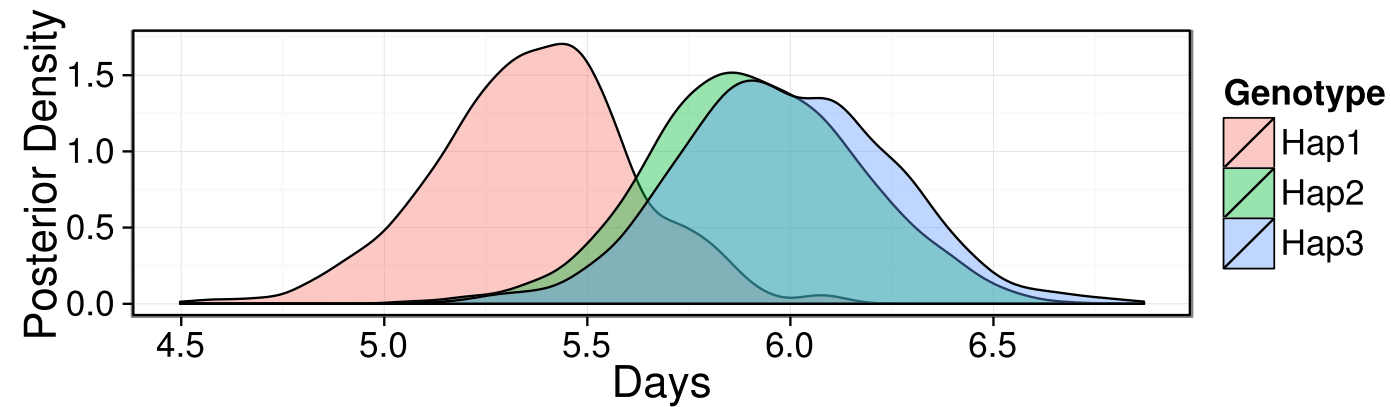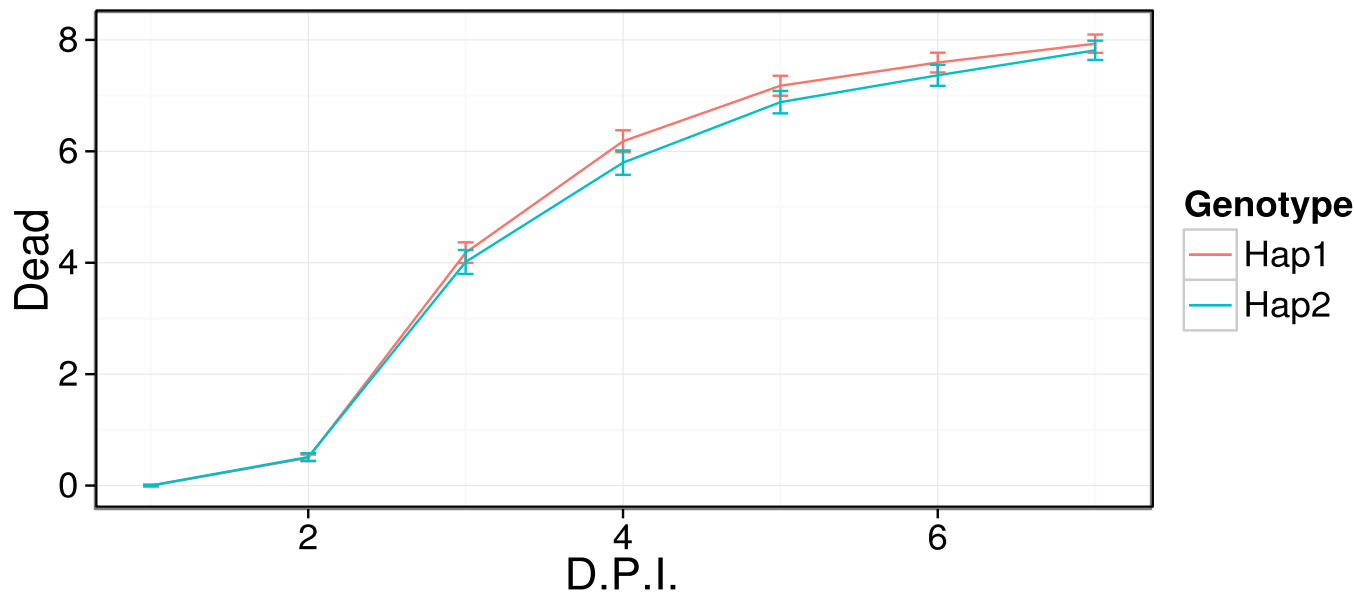

Supplement: Supplemental Material [file supp_g3.116.031880_FigureS7.pdf]
